# Annatto (*Bixa orellana*) δ-TCT Supplementation Protection against Embryonic Malformations through Alterations in PI3K/Akt-Cyclin D1 Pathway

**DOI:** 10.3390/biom9010019

**Published:** 2019-01-10

**Authors:** Siti Syairah Mohd Mutalip, Mohd Hamim Rajikin, Sharaniza Ab Rahim, Nor-Ashikin Mohamed Noor Khan

**Affiliations:** 1 Faculty of Pharmacy, Universiti Teknologi MARA (UiTM) Puncak Alam Campus, Selangor 42300, Malaysia; 2 Maternofetal and Embryo Research Group (MatE), Universiti Teknologi MARA (UiTM), Selangor 40450, Malaysia; 3 Faculty of Medicine, Universiti Teknologi MARA (UiTM) Sg. Buloh Campus, Selangor 47000, Malaysia; hamim400@salam.uitm.edu.my (M.H.R.); sharaniza_abrahim@salam.uitm.edu.my (S.A.R.); noras011@salam.uitm.edu.my (N.A.M.N.K.)

**Keywords:** murine preimplantation embryos, vitamin E, annatto delta-tocotrienol, tocopherols, reproductive toxicant, nicotine

## Abstract

Protective action by annatto-derived delta-tocotrienol (δ-TCT) and soy-derived alpha-tocopherol (α-TOC) through the regulation of the PI3K/Akt-cyclin D1 pathway against nicotine-induced DNA damage is the focus of the present study. Nicotine, which has been widely reported to have numerous adverse effects on the reproductive system, was used as a reproductive toxicant. 48 female balb/c mice (6–8 weeks) (23–25 g) were randomly divided into eight groups (Grp.1–Grp.8; *n* = 6) and treated with either nicotine or/and annatto δ-TCT/soy α-TOC for seven consecutive days. On Day 8, the females were superovulated and mated before euthanization for embryo collection (46 h post-coitum). Fifty 2-cell embryos from each group were used in gene expression analysis using Affymetrix QuantiGene Plex2.0 assay. Findings indicated that nicotine (Grp.2) significantly decreased (*p* < 0.05) the number of produced 2-cell embryos compared to the control (Grp.1). Intervention with mixed annatto δ-TCT (Grp.3) and pure annatto δ-TCT (Grp.4) significantly increased the number of produced 2-cell embryos by 127% and 79%, respectively compared to Grp.2, but these were lower than Grp.1. Concurrent treatment with soy α-TOC (Grp.5) decreased embryo production by 7%. Supplementations with δ-TCT and α-TOC alone (Grp.6-Grp.8) significantly increased (*p* < 0.05) the number of produced 2-cell embryos by 50%, 36%, and 41%, respectively, compared to control (Grp.1). These results were found to be associated with alterations in the PI3K/Akt-Cyclin D1 genes expressions, indicating the inhibitory effects of annatto δ-TCT and soy α-TOC against nicotinic embryonic damage. To our knowledge, this is the first attempt in studying the benefits of annatto δ-TCT on murine preimplantation 2-cell embryos.

## 1. Introduction

The Phosphatidylinositol-3-kinase (PI3K)/Akt-Cyclin D1 signaling pathway has been reported to be involved in the regulation of embryonic cell proliferation in human and animals. This pathway plays important roles in the regulations of cell growth, survival (anti-apoptosis), development, glucose metabolism, and glycogen biosynthesis [1,2,3,4]. The involvement of this pathway in human and animal preimplantation embryos has been previously reported [5,6,7,8,9,10,11,12,13,14,15,16] and it is suggested that its inhibition could lead to truncated embryonic development. In the context of reproductive toxicology, the adverse effects of nicotine as one of the reproductive toxicants have been extensively reported in human [17,18] and animal [19,20,21,22,23] studies. The exposure of nicotine to the body system is most commonly caused by cigarette smoking. According to Dechanet et al. [24], cigarette smoke contains thousands of harmful substances, including nicotine, polycyclic aromatic hydrocarbons, cadmium, and many more. These carcinogenic substances have a high potential for exerting negative effects at each stage of reproductive function, including folliculogenesis, steroidogenesis, embryo transport, endometrial receptivity, endometrial angiogenesis, uterine blood circulation, and uterine myometrium [24]. Besides these effects, the nicotinic activation of the PI3K/Akt pathway was also reported to contribute to the growth of a number of cancer types [25,26].

Vitamin E is a lipid-soluble substance [27,28] that contains two major elements; tocotrienols (TCTs) and tocopherols (TOCs). Both elements are present in four different homologs, known as α-tocotrienol, β-tocotrienol, γ-tocotrienol, δ-tocotrienol, α-tocopherol, β-tocopherol, γ-tocopherol, and δ-tocopherol [29]. Vitamin E was first discovered in 1922 as an important substance that is needed for fertility [30]; however over the decades, it became well recognized as an antioxidant, following extensive study reports [31,32,33]. The benefits of vitamin E, especially TCTs as antioxidant and anticancer agents have been reported earlier [31,32,33,34,35]. Besides, TCTs were also reported to exhibit anti-proliferative [36], anti-survival [37], pro-apoptotic [38], anti-angiogenic [39], and anti-inflammatory [40] activities. Despite these numerous available reports, studies on the effects of TCTs on reproductive health, particularly on fertility, sterility and preimplantation embryonic development remains largely unknown, with only a few attempts being made recently [21,23,41,42,43].

Vitamin E is available in various foods and plants, ranging from edible oils to nuts, including wheat, rice bran, barley, oat, coconut, palm, and annatto [44,45]. In general, the sources of vitamin E, such as palm oil and rice bran, contain approximately between 25–50% of the α-TOC homolog in the total vitamin E content. In contrast, TCTs derived from annatto (*Bixa orellana*) seeds were discovered to not contain any tocopherol (TOC) homologs [46]. The ‘tocopherol-free’ aspect of annatto-TCTs is highly valuable, since previous studies have shown that α-TOC interferes with the benefits of TCTs [47,48]. The advantage of having the ‘only-TCT’ derivative makes annatto the first and only true source of naturally derived vitamin E that supplies only TCT to-date [46]. The additional discovery of annatto-TCT containing only γ- and δ-homologs also makes it the only known source of tocotrienol that provides the highest content of the powerful δ-tocotrienol [46].

Taking together the nicotinic adverse effects and the potential of vitamin E on reproductive functions, the present study was conducted to investigate the protective effects of annatto δ-TCT through the regulation of the PI3K/Akt-Cyclin D1 pathway against nicotine-induced DNA damage in murine preimplantation 2-cell embryos. The effects of annatto δ-TCT on PI3K/Akt-Cyclin D1 regulation in normal embryos of nicotine non-treated mice were also studied.

## 2. Materials and Methods

### 2.1. Ethics Approval

**Figure 1:**
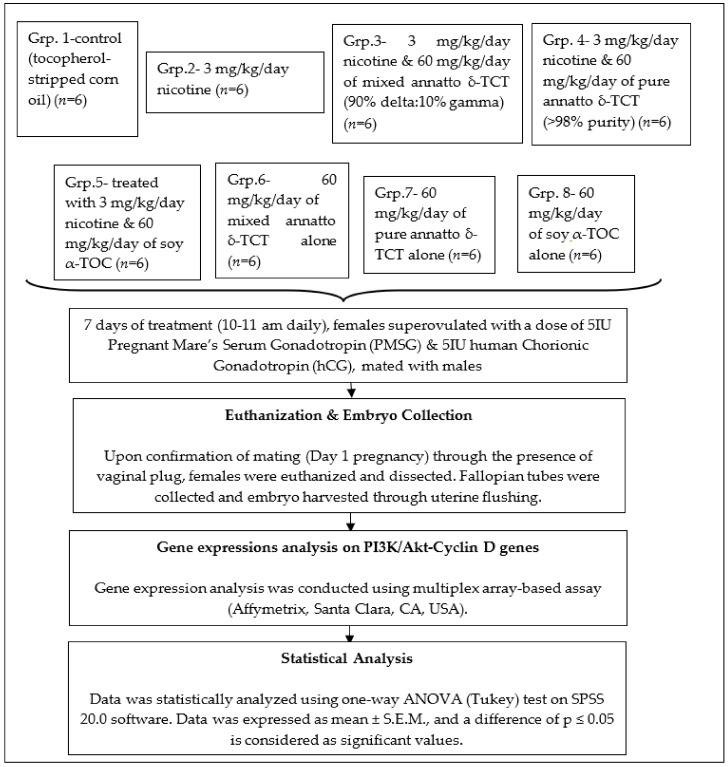
The overall study design as employed in the present research.

This study has been approved by the university’s Committee on Animal Research and Ethics (CARE) and Animal Care and Use Committee (ACUC-7/13). The overall study design is shown in Figure 1.

### 2.2. Animal Treatment

Forty-eight (48) males and 48 female balb/c mice aged 6–8 weeks (20–25 g) were obtained (Chenur Supplier, Selangor, Malaysia). All animals were acclimatized for a week and maintained at a controlled temperature and humidity (24 °C, 12-h light/dark cycle). The animals were fed with purchased vitamin E-free pellets (Gold Coin Holdings, Kuala Lumpur, Malaysia), and water was given ad libitum. The extract samples of annatto (*Bixa orellana*) δ-TCT in both mixed form (containing 90% delta and 10% gamma homologs) and pure form (contains more than 98% delta homologs) were provided by American River Nutrition Inc. (ARN), Hadley, MA, United States of America (USA). Samples of alpha-tocopherol (α-TOC) derived from soy bean were also provided by ARN. The preparation of samples involved mixing the extract samples to the tocopherol-stripped corn oil (vehicle).

All females were randomly divided into eight groups (Grp.1–Grp.8) with six mice each (*n* = 6). Those groups were assigned according to the following treatments (Table 1) for seven consecutive days. Briefly, the treatments were: Grp.1 (control)—given 0.1 mL tocopherol-stripped corn oil (Acros, Belgium) (oral gavage), Grp.2—given 3 mg/kg/day of nicotine (s. c. injection), Grp.3—concurrently treated with 3 mg/kg/day nicotine (s. c. injection) and 60 mg/kg/day of mixed δ-TCT (oral gavage), Grp.4—concurrently treated with 3 mg/ kg/day nicotine (s. c. injection) & 60 mg/kg/day of pure δ-TCT (>98% purity) (oral gavage), Grp.5—concurrently treated with 3 mg/kg/day nicotine (s. c. injection) & 60 mg/kg/day of α-TOC (oral gavage), Grp.6—given 60 mg/ kg/day of mixed δ-TCT alone (oral gavage), Grp.7—given 60 mg/kg/day of pure δ-TCT alone (oral gavage) and Grp.8—given 60 mg/kg/day α-TOC alone (oral gavage). Females were treated at between 10:00–11:00 a.m. daily. This experiment was conducted in three replicates.

### 2.3. Superovulation

Upon completion of seven days of treatment, all the females were injected subcutaneously (s. c.) with 5 IU of pregnant mare’s serum gonadotropin (PMSG) (Sigma Aldrich, St. Louis, MO, USA) between 10:00 a.m. to 11:00 a.m. (on Day 8) and left for 48 h. PMSG was used to mimic the oocyte maturation effect of the endogenous follicle-stimulating hormone (FSH) [49]. After 48 h, the females were injected s.c. with 5 IU of human chorionic gonadotropin (hCG) (Sigma Aldrich, St. Louis, MO, USA), also between 10:00 a.m. to 11:00 a.m. (on Day 10) and were immediately subjected for mating. hCG was used to mimic the ovulation induction effect of luteinizing hormone (LH), and it functions to promote the maintenance of the corpus luteum during the beginning of pregnancy [50].

### 2.4. Mating

All males and superovulated females (immediately after hCG injection) were arranged for mating in the formation of 1:1, and housed in a cage for 48 h. Mating was confirmed by the presence of a vaginal plug. Females were euthanized at 46 h post-coitum by cervical dislocation between 8:00 and 9:00 a.m. (Day 12).

### 2.5. Embryo Collections and Culture

Euthanized females were immediately dissected to retrieve the Fallopian tubes, which were flushed with M2 medium (Sigma Aldrich, St. Louis, MO, USA) for embryo collection under a dissecting microscope (Leica Zoom 2000, Tokyo, Japan). Collected embryos were graded according to the quality of the embryos [51]. Normal with good quality embryos were washed again in M2 medium before being cultured in 100 μL M16 culture medium (Sigma Aldrich, St. Louis, MO, USA) and overlaid with mineral oil (Sigma Aldrich, St. Louis, MO, USA). Culture medium was prepared overnight for homogenization prior to use. Embryos were incubated overnight under 5 % CO_2_ at 37 °C. This procedure was done following the standard protocol of embryo handling, as described by [50].

### 2.6. Gene Expression Analysis

Fifty 2-cell embryos (*n* = 50) from each group (Grp.1–Grp.8) of each replicate were collected and kept in 100 μL cryopreservation media (Gibco, Invitrogen Ltd., Paisley, UK) at –20 °C until they were used in gene expression analysis. The analysis was conducted using QuantiGene Plex assay (QGP) (Affymetrix, Santa Clara, CA, USA) at i-DNA Biotechnology (M) Sdn. Bhd., Kuala Lumpur, Malaysia. The procedures were followed as described in the manufacturer’s protocol. The advantage of using the QGP method is that it allows for mRNA quantification directly from the embryonic cell lysate through sequence-specific probe-gene hybridization without the necessity of extracting RNA. This reduces the errors that might be possibly introduced onto the samples during the RNA extraction and amplification procedures [52,53,54,55,56].

The present study focused on the PI3K/Akt-Cyclin D1 pathway and the analyzed genes were: *Pik3ca* (Accession No.: NM_008839), *Pik3cb* (Accession No.: NM_029094), *Pdpk1* (Accession No.: NM_011062), *Akt1* (Accession No.: NM_009652), *PTEN* (Accession No.: NM_008960), *GSK3β* (Accession No.: NM_019827), *ATM* (Accession No.: NM_007499), *Ccnd1* (Accession No.: NM_007631), *Ccne1* (Accession No.: NM_007633), *Cdk2* (Accession No.: NM_016756), *Cdk4* (Accession No.: NM_009870), *Cdk6* (Accession No.: NM_009873), *Cdkn1a* (Accession No.: NM_007669), *Cdkn1b* (Accession No.: NM_009875) and *Trp53* (Accession No.: NM_011640); and reference genes *Hprt1* (Accession No.: NM_013556), *Gapdh* (Accession No.: NM_008084), and *Actb* (β-actin) (Accession No.: NM_007393). The obtained raw data were the median fluorescence intensity (MFI) values, normalized against the hypoxanthine–guanine phosphoribosyltransferase 1 (*Hprt1*) gene and the control value, to obtain the relative fold-change (FC) value.

### 2.7. Statistical and Pathway Analysis

Gene Network Central Pro (GNCPro) (http://gncpro.sabi-osciences.com/gncpro/gncpro.php) software was used to view the interactions, and to analyze the relationship between the PI3K/Akt-Cyclin D1 genes.

Data on the numbers of obtained 2-cells embryos and the FC values from gene expression analysis were statistically compared between the treatments and their respective controls. Equality of variance was analyzed by using Levene’s Test, followed by one-way ANOVA with a post hoc (Tukey) test. Data normality was determined using the Shapiro–Wilk test. Data were expressed as mean ± SEM, and a *p*-value of < 0.05 was considered to be statistically significant. Analyses were done using SPSS software (version 20).

## 3. Results

### 3.1. Embryo Production and Retrieval

**Figure 2:**
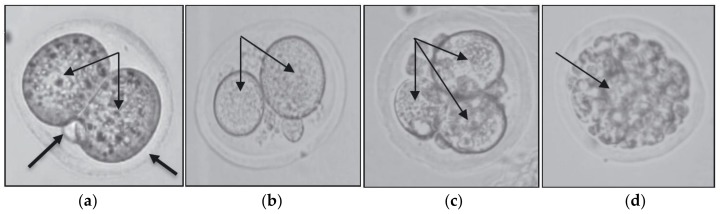
(**a**) Normal 2-cell embryos obtained in Grp.1, Grp.6, Grp.7, and Grp.8. Normal appearances are characterized by the equal blastomere size (double arrows), normal zona pellucida (ZP) lining, and presence of a polar body (thick arrows). Abnormal 2-cell embryos obtained in Grp.2, Grp.3, Grp.4, and Grp.5 is shown in (**b**–**d**). Abnormal appearances are characterized by (**b**) unequal blastomere size, (**c**) asymmetrical blastomere division, and (**d**) blastomere fragmentations.

Normal and abnormal appearances of 2-cell embryos collected from the experimental groups are shown in Figure 2. Table 2 summarized the average number of the produced 2-cell embryos in each mouse of every group. One-way ANOVA analysis showed that the mean number of embryos produced following nicotine treatment (Grp.2) was significantly lower than the control group (Grp.1). Intervention with mixed δ-TCT (Grp.3) and pure δ-TCT (Grp.4) significantly (*p* < 0.05) increased the number of produced 2-cell embryos by 127 % (Grp.3) and 79 % (Grp.4) compared to G2; however, they were considerably lower than the control value (Grp.1). Furthermore, concurrent treatment with α-TOC reduced the number of produced 2-cell embryos by 7 %. In contrast, supplementation with mixed δ-TCT (Grp.6), pure δ-TCT (Grp.7), and α-TOC (Grp.8) alone resulted in a significant increase (*p* < 0.05) in the mean number of 2-cell embryos by 50 %, 36 %, and 41 % respectively, in comparison to the control group (Grp.1).

### 3.2. Gene Expression Analysis

Data on the fold-change value of the studied genes are shown in Table 3. The present findings indicated that following treatment with 3 mg/kg/day of nicotine in Grp.2, the *pik3cb*, *PTEN*, *pdpk1*, *akt1*, *cdk4*, *cdkn1a* and *cdkn1b* genes were down-regulated, whereas the other genes showed non-significant changes in the fold-change value (Table 3). These results following nicotine treatment on the gene expressions might explain the failure of embryonic development in this group (Grp.2), as the retrieved numbers of 2-cell embryos were significantly lower than the retrieved numbers of embryos in the control group (Grp.1) (Table 2).

Intervention with δ-TCT in Grp.3 and Grp.4 both resulted in a significant up-regulation of the *pik3cb* gene, at 1.92-fold in Grp.3 and 2.56-fold in Grp.4. This was further followed by a significant up-regulation, 13.46-fold (Grp.3) and 17.92-fold (Grp.4), of the *PTEN* gene. However, the expressions of PDK1 (*pdpk1*) gene in both groups were down-regulated. A similar pattern of expression was observed in the cell cycle genes. The major cell proliferation regulators, including *atm* and *trp53*; and the cyclin-dependent kinase inhibitor genes (CDKI)—*cdkn1a* and *cdkn1b*, were all significantly up-regulated in both Grp.3 and Grp.4 (Table 3). Cyclins and cyclin-dependent kinases (Cyclin E1-CDK2) were also significantly up-regulated in both groups (Table 3).

Concurrent supplementation of nicotine with α-TOC in Grp.5 resulted in up-regulations of *pik3cb*, *PTEN*, and *pdpk1* at 4.28-fold, 17.92-fold, and 1.2-fold, respectively. Treatment with α-TOC also resulted in the significant up-regulation of *atm*, *cdkn1a*, *cdkn1b*, and *trp53*. Cyclin E1 gene (*ccne1*) and *cdk2* were also significantly up-regulated at 16.75- and 41.75-fold, respectively (*p* < 0.05) (Table 3).

Supplementation with mixed and pure δ-TCT alone in Grp.6 and Grp.7 resulted in different expression patterns of the studied genes. *Pik3ca* and *PTEN* expressions were down-regulated at 0.33- and 0.61-fold respectively, with *pik3cb* remained unchanged in Grp.6. *Pdpk1* (PDK1) and *akt1* were significantly up-regulated. The other genes (*GSK3β*, *atm*, *ccnd1*, *ccne1*, *cdk2*, *cdk4*, *cdk6*, *atm*, *cdkn1a*, *cdkn1b*, and *trp53*) were either down-regulated or non-significantly changed. Treatment with pure δ-TCT alone (Grp.7) resulted in an unchanged expression of *pik3ca*, *pik3cb*, and *PTEN*, whereas *pdpk1* and *akt1* were significantly up-regulated, at 1.41-fold and 1.48-fold, respectively (*p* < 0.05). The other genes were all down-regulated, except the genes *GSK3β*, *atm*, and *trp53* were non-significantly changed. Meanwhile, supplementation with α-TOC alone (Grp.8) resulted in the up-regulation of *pdpk1* and *akt1*, at 1.64-fold and 1.35-fold increase respectively, while the other genes were either down-regulated or non-significantly changed (Table 3).

### 3.3. Pathway Analysis

**Figure 3:**
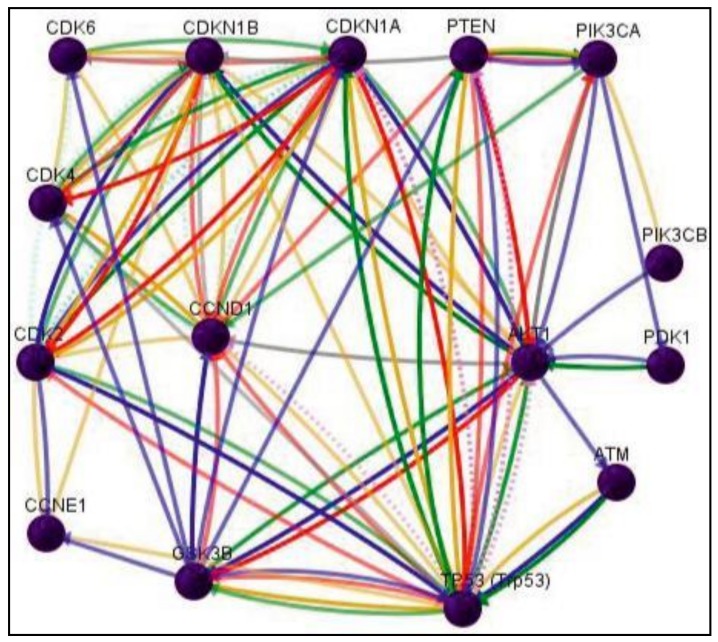
Molecular interactions between the studied genes. *The lines represent the down-regulation (red), up-regulation (green), functional and transcriptional regulation (grey), co-expression (purple), chemical modification (phosphorylation, post-transcriptional modifications, etc.) (blue), physical interaction (yellow), predicted protein interaction (pale-blue dotted line), and predicted transcription factor regulation (purple dotted line). Abbreviations: Akt1-V-Akt Murine Thymoma Viral Oncogene-Like Protein 1; Atm-Ataxia Telangiectasia Mutated; Ccnd1-Cyclin D1; Ccne1-Cyclin E1; Cdk2-Cyclin Dependent Kinase 2; Cdk4-Cyclin Dependent Kinase 4; Cdk6-Cyclin Dependent Kinase 6; Cdkn1a-Cyclin Dependent Kinase Inhibitor 1A; Cdkn1b-Cyclin Dependent Kinase Inhibitor 1B; GSK3β-Glycogen Synthase Kinase 3 Beta; Pdpk1–3-Phosphoinositide Dependent Protein Kinase 1; Pik3ca-Phosphatidylinositol–4,5-Bisphosphate 3-Kinase Catalytic Subunit Alpha; Pik3cb-Phosphatidylinositol–4,5-Bisphosphate 3-Kinase Catalytic Subunit Beta; PTEN-Phosphatase And Tensin Homolog Deleted On Chromosome 10; Trp53-Transforma-tion Related Protein 53.

**Figure 4:**
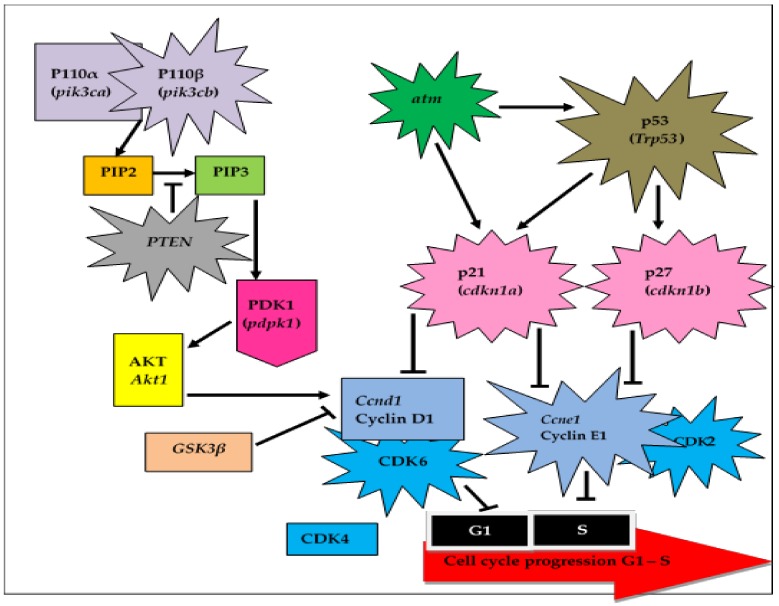
Alterations of the PI3K/Akt-Cyclin D1 pathway in cell cycle progression from G1 to S phase in 2-cell embryos. Concurrent treatment with nicotine and mixed/pure δ-TCT (Grp.3 & Grp.4) resulted in the upregulation of *pik3cb* and *PTEN*. These increments attenuate the conversion of PIP2 to PIP3 substance by *PTEN*. A low abundance of PIP3 will reduce the rate of PDK1 activation. Although supplemented with δ-TCT, the detection of DNA damage following nicotine treatment increased the expression of the checkpoint gene, *atm*. High level of *atm* will phosphorylate (activate) its target gene, *trp53*. Activated p53 binds and activates CDKIs (p21 and p27). Both CDKIs bind and suppress the overexpression of cyclin E1/CDK2 complex, which will cause disruption in the G1/S transition. This will eventually mitigate the entire embryonic cell proliferation. Abbreviations: Akt1-V-Akt Murine Thymoma Viral Oncogene-Like Protein 1; Atm-Ataxia Telangiectasia Mutated; Ccnd1-Cyc-lin D1; Ccne1-Cyclin E1; Cdk2-Cyclin Dependent Kinase 2; Cdk4-Cyclin Dependent Kinase zt; Cdk6-Cyclin Dependent Ki-nase 6; Cdkn1a-Cyclin Dependent Kinase Inhibitor 1A; Cdkn1b-Cyclin Dependent Kinase Inhibitor 1B; GSK3β-Glycogen Synthase Kinase 3 Beta; Pdpk1–3-Phosphoinositide Dependent Protein Kinase 1; Pik3ca-Phosphatidylinositol–4,5-Bisphos-phate 3-Kinase Catalytic Subunit Alpha; Pik3cb-Phosphati-dylinositol–4,5-Bisphosphate 3-Kinase Catalytic Subunit Beta; PTEN-Phosphatase And Tensin Homolog Deleted On Chromosome 10; Trp53-Transformation Related Protein 53.

Pathway analysis using Gene Network Central Pro (GNCPro) confirmed the close interactions and high functional influences (as defined by links to peer-reviewed publications) of the studied PI3K/Akt-Cyclin D1 genes (Figure 3). Subsequently, schematic models have been drawn as an attempt to explain how the changes in the studied genes (based on the data from Table 3) following interventions with mixed δ-TCT and pure δ-TCT on nicotine-treated mice would influence the fate of the embryonic cells (Figure 4).

## 4. Discussion

The present data showed that nicotine significantly decreased (*p* < 0.05) the number of retrieved embryos in Grp.2 (Table 2). This result is in line with previous studies on the effects of nicotine on oocytes and embryos, which reported that nicotine adversely affects the structures of oocytes [21], the rate of embryonic cleavage [57,58], the number of retrieved embryos [20], the number of hatched blastocysts, and the rate of implantation [23,59]. In this present study, the nicotinic damaging effects in Grp.2–Grp.5 are clearly seen, as shown in Figure 2. With the results of abnormal embryonic physical appearances and low numbers of retrieval, it is speculated that nicotine might have exerted its adverse effects (damage) to the oocytes prior to fertilization, and caused embryonic DNA damage, resulting in the poor 2-cell embryo retrieval in Grp.2. This was further explained by the downregulation and inhibition of PI3K/Akt-Cyclin D1 genes in Grp.2 (Table 3).

Concurrent treatments with nicotine and annatto δ-TCT resulted in significant (*p* < 0.05) increases of 127 % and 79 % in Grp.3 and Grp.4, respectively, in the mean number of produced 2-cell embryos (Table 2) (compared to Grp.2). However, these increases were considerably low compared to the numbers of 2-cells embryos obtained from the control group (Grp.1). Results found that the changes in the embryo retrieval were influenced by the alterations in PI3K/Akt-Cyclin D1 pathway in Grp.3 and Grp.4 (Figure 4). It is suggested that maternal supplementations of annatto δ-TCT delayed the proliferation of damaged embryonic cells through the upregulation of major cell cycle checkpoint regulators, including tumor suppressor genes *PTEN*, *atm*, and *trp53*; as well as the CDKIs p21 and p27, although the overexpression of cyclin E1/CDK2 complex promotes the G1/S phase transition (Table 3, Figure 4). These showed that annatto δ-TCT delayed embryonic growth through their anti-proliferative effects, which resulted in a poor rate of embryonic development (in comparison to Grp.3 and Grp.4 to Grp.1).

Overexpression of cyclin E1/CDK2 is a biomarker of uncontrolled cell growth following DNA damage. An earlier study using fibroblasts constructed to constitutively express human cyclin E reported that cyclin E overexpression shortened the duration of G1, decreased the cell size and reduced the serum required for the G1/S phase transition [60]. Overexpression of the cyclin E1/CDK2 complex also has been reported in immortalized rat embryo fibroblasts [61] and breast cancer cells [62]. Although it seems to be an improvement in the numbers of produced 2-cell embryos in Grp.3 & Grp.4 compared to Grp.2, the expressions of the studied genes were highly affected. This suggests that the anti-proliferative effects of annatto δ-TCT that targeted the major cell cycle checkpoint regulators in the damaged embryonic cells in Grp.3 and Grp.4.

The anti-proliferative effects of annatto δ-TCT against DNA damage, as shown in the present study are in line with previous reports conducted using δ-TCT. A recent study on the effects of dietary supplementation with mixed annatto-TCT (90% δ-TCT and 10% γ-TCT) on the spontaneous development of mammary tumors in HER-2/neu transgenic mice resulted in the delayed development of mammary tumors, and a reduction of the number and size of mammary tumor masses and those of lung metastases. In annatto-TCT-supplemented mice, both apoptosis and senescent-like growth arrest of tumor cells were increased in mammary glands [63]. Constantinou et al. [64] reported that hypomethylated configurations of TCT (HM-TCT), which are γ-TCT and δ-TCT homologs, represent the most potent anti-cancer forms of vitamin E in in vitro studies. Anti-cancer (anti-proliferative) effects of δ-TCT against pancreatic adeno-carcinoma cells [65] and γ-TCT against colon cancer [66] and melanoma cells [40] also have been reported. Nesaretnam et al. [67] reported on the anti-proliferative effect of both γ-TCT and δ-TCT in estrogen-sensitive (MCF-7 or ZR-75–1) and insensitive (MDA-MB 435 or 231) human breast cancer cells. Another study using human breast cancer cells reported that δ-TCT inhibited the cell cycle through the control of Rb/cyclin D/CDK4 pathway, and induced apoptosis [68]. The anticancer effects of both γ-TCT and δ-TCT through various mechanisms have been also reported [69].

The mean number of 2-cell embryos in Grp.5 was significantly (*p* < 0.05) decreased by 7%. The decrement was also suggested to be associated with alterations in PI3K/Akt-cyclin D1 pathway. Present data showed that almost all of the genes were upregulated in Grp.5 (Table 3). As seen in Grp.3 & Grp.4, the major cell cycle checkpoint regulators, including tumor suppressor genes *PTEN*, *atm* and *trp53*; as well as CDKIs (p21 and p27) were also highly upregulated in this group. In addition, these were followed by a decrease in the expression of *akt1* by 0.45-fold, and an increase of *GSK3β* by 1.07-fold (Table 3). The upregulations of the major cell cycle check-point regulators function to arrest cell proliferation. It is suggested that together with these, a slight upregulation of *GSK3β* may influence the control on the proteolytic degradation of cyclin D1 through the induction of the nuclear to cytoplasm translocation [70,71]. The expression of cyclin E was also increased, but it was relatively lower than in Grp.3 and Grp.4. Therefore, all of these upregulations might explain the detrimental effect of α-TOC in 2-cell embryos against the nicotine effects (Table 2). The present results on the detrimental effect of α-TOC are in agreement with previous reports on the anti-proliferative effect of this vitamin E homolog. From earlier reports on the ability of α-TOC to prevent the formation of cancer-promoting nitrosamine in the stomach [72,73], up to the recent report on the role of α-TOC as an anti-inflammatory and antioxidant agent that protected the colonic mucosa injury in induced colitis in rats [74], the anti-proliferative effect of α-TOC has been extensively studied and reported in a number of diseases.

Data on the mean number of the produced 2-cell embryos showed significant increases (*p* < 0.05) following treatments with annatto δ-TCT (Grp.6 and Grp.7) and α-TOC (Grp.8) alone. The increases were due to the slight changes in PI3K/Akt-Cyclin D1 gene expression. The present data showed that in Grp.6–Grp.8, almost all of the genes were in normal expression ratios. The tumor suppressor genes *PTEN*, *atm*, and *trp53*; and CDKIs (p21 and p27) were either downregulated or unchanged. The low presence of these genes supports cell proliferation through the normal G1/S transition. This was followed by an increase in PDK1 and *akt1* genes in Grp.6—Grp.8 (Table 3). It is suggested that the increase of PDK1 and *akt1*, following treatment with annatto δ-TCT and α-TOC enhanced the pathway regulation, leading to an increase in the proliferation rate and the number of retrieved 2-cell embryos (Table 2). Cyclin D1-CDK4/6 and cyclin E1/CDK2 were also present at low expression, a condition that facilitates the normal progression of G1/S transition in preimplantation mouse embryos [75].

The present study provided evidence for two different functions of annatto δ-TCT and α-TOC in two different conditions. In stress-induced environments (following nicotine injection), these types of vitamin E tend to function as a protectant, by suppressing the 2-cell murine preimplantation embryos from growing. Whereas, in stress-free conditions (normal), these types of vitamin E played a role in promoting 2-cell murine preimplantation embryonic growth.

Based on our knowledge, this study is the first attempt to study the regulation of the PI3K/Akt-Cyclin D1 pathway by annatto δ-TCT and soy α-TOC on 2-cell murine preimplantation embryos under both normal and abnormal conditions. Maternal antioxidants levels were not measured, since the focus of this study was to understand the changes in PI3K/Akt-cyclin D1 regulation, following the given treatments. In addition, the used experimental dosages were considered from previous studies using palm-tocotrienol-rich fractions (TRF) (containing all the α-, β-, γ- and δ-TCT and α-TOC) which showed that a dose of 30 mg/kg/day is optimal for embryonic growth following nicotine treatment [23]. Another study on the beneficial effects of palm-TRF supplementations on pregnancy outcome in corticosterone (CORT)-treated mice showed that the optimum dose of TCT that is able to overcome the effects of CORT on the numbers of implantation sites and resorption rate was 120 mg/kg/day [42]. Taking the results from previous studies [23,42], 60 mg/kg/day of annatto δ-TCT was intended to be used in the present study as the moderate dosage to achieve the same effects as palm-TRF.

## 5. Conclusions

In conclusion, the present study showed novel findings on the ability of annatto δ-TCT in providing promising effects on embryonic development, which might serve as an alternative to α-TOC.

## Figures and Tables

**Table 1:** Treatments given to the experimental groups for seven consecutive days.

Groups	Treatment	Route of Administration
Grp.1 (control) (*n* = 6)	Corn oil tocopherol-stripped (0.1 mL) (vehicle)	Oral-gavage
Grp.2 (*n* = 6)	Nicotine (3 mg/kg/day)	Subcutaneous (s. c.) injection
Grp.3 (*n* = 6)	Nicotine (3 mg/kg/day) and mixed δ-TCT (90% delta:10% gamma) (60 mg/kg/day)	Subcutaneous injection (s. c.) & oral gavage (Mixed δ-TCT, pure δ-TCT and α-TOC were dissolved in tocopherol-stripped corn oil prior to force-feeding)
Grp.4 (*n* = 6)	Nicotine (3 mg/kg/day) and pure δ-TCT (delta > 98% purity) (60 mg/kg/day)	
Grp.5 (*n* = 6)	Nicotine (3 mg/kg/day) and α-TOC (60 mg/kg/day)	
Grp.6 (*n* = 6)	Mixed δ-TCT alone (60 mg/kg/day)	Oral-gavage (Mixed δ-TCT, pure δ-TCT and α-TOC were dissolved in tocopherol-stripped corn oil prior to force-feeding)
Grp.7 (*n* = 6)	Pure δ-TCT alone (60 mg/kg/day)	
Grp.8 (*n* = 6)	α-TOC alone (60 mg/kg/day)	

* δ-TCT = delta tocotrienol (annatto-derived); α-TOC = alpha tocopherol (soy-derived).

**Table 2:** Average number of 2-cell embryos produced per mice per group (*n* = 6).

Treatment Groups	Mean + SEM
Grp.1—Corn oil tocopherol stripped	22.11 ± 0.40
Grp.2—Nicotine (3 mg/kg/day)	7.28 ± 0.29 ^a^
Grp.3—Nicotine and δ-TCT mixed	16.56 ± 0.39 ^b^
Grp.4—Nicotine and pure δ-TCT	13.06 ± 0.38 ^b^
Grp.5—Nicotine and α-tocopherol	6.78 ± 0.30
Grp.6—δ-TCT mixed alone	33.17 ± 0.28 ^a^
Grp.7—Pure δ-TCT alone	30.11 ± 0.29 ^a^
Grp.8—α-tocopherol alone	31.22 ± 0.27 ^a^

^a^ Indicates statistically significant change (*p* < 0.05) in comparison to Grp.1. ^b^ Indicates statistically significant change (*p* < 0.05) in comparison to Grp.2.

**Table 3:** Fold change values of the PI3K/Akt-Cyclin D1 genes of each group (*n* = 6).

Genes	Grp.1	Grp.2	Grp.3	Grp.4	Grp.5	Grp.6	Grp.7	Grp.8
*Pik3ca*	1	–1.2	–1.75	–1	–1	γ0.33 **	1	γ0.84 **
*Pik3cb*	1	γ0.91	ϕ1.92 *	ϕ2.56 *	ϕ4.28 *	1	1	ϕ0.93
*PTEN*	1	γ0.18 **	ϕ13.46*	ϕ17.92 *	ϕ17.92 *	γ0.61 **	1	γ0.25 **
*Pdpk1*	1	γ0.34 **	γ0.3 **	γ0.4 **	ϕ1.2 *	ϕ1.59 *	ϕ1.41 *	ϕ1.64 *
*Akt1*	1	γ0.53 **	–0.74	–1.31	γ0.45 **	ϕ2.41 *	ϕ1.48 *	ϕ1.35 *
*GSK3β*	1	–1.04	–6.73	–6.42	ϕ1.07	–1.72	–0.56	–3.28
*Atm*	1	–0.13	ϕ5.77 *	ϕ7.69 *	ϕ2.54 *	–0.43	–0.15	–0.18
*Ccnd1*	1	–0.04	–12.5	–8.25	–8.25	γ0.43 **	γ0.43 **	γ0.45 **
*Ccne1*	1	–0.04	ϕ43.75 *	ϕ41.75 *	ϕ16.75 *	γ0.43 **	γ0.53 **	γ0.27 **
*Cdk2*	1	–0.04	ϕ31.25 *	ϕ25 *	ϕ41.75 *	γ0.45 **	γ0.35 **	γ0.27 **
*Cdk4*	1	γ0.13 **	–5.77	–2.54	ϕ5.15 *	γ 0.43 **	γ0.15 **	γ0.65 **
*Cdk6*	1	–0.04	ϕ18.75 *	ϕ8.25 *	ϕ8.25 *	γ 0.43 **	γ0.43 **	γ0.09 **
*Cdkn1a*	1	γ0.65 **	ϕ5.77 *	ϕ5.65 *	ϕ6.66 *	–0.14	γ0.17 **	–1.11
*Cdkn1b*	1	γ0.3 **	ϕ1.83 *	ϕ5.57 *	ϕ11.1 *	–0.15	γ0.14 **	–0.09
*Trp53*	1	–0.04	ϕ18.75 *	ϕ16.75 *	ϕ17.55 *	–0.14	–0.43	γ0.27 **

Groups: Grp.1 serves as a control, and received 0.1 mL of tocopherol stripped corn oil, Grp.2 was given 3 mg/kg/day of nicotine, Grp.3 was concurrently treated with 3 mg/kg/day nicotine and 60 mg/kg/day of mixed δ-TCT (90% delta:10% gamma), Grp.4 was concurrently treated with 3 mg/kg/day nicotine and 60 mg/kg/day of pure δ-TCT (>98% purity), Grp.5 was concurrently treated with 3 mg/kg/day nicotine and 60 mg/kg/day of α-TOC, Grp.6 received 60 mg/kg/day of mixed δ-TCT alone, Grp.7 received 60 mg/kg/day of pure δ-TCT alone, and Grp.8 received 60 mg/kg/day α-TOC alone. Mode of regulation is indicated based on the reference value of one (1). Values of less than 1 (γ) = downregulated genes; more than 1 (ϕ) = upregulated genes; non-significant values (negative (−) values) and the value ‘1’ = unchanged value of gene expressions ratio. * Indicates significant (*p* < 0.05) increase in the fold change value (corresponds to upregulation). ** Indicates significant (*p* < 0.05) decrease in the fold change value (corresponds to downregulation). Abbreviations: *Akt1*-V-Akt Murine Thymoma Viral Oncogene-Like Protein 1; *Atm*-Ataxia Telangiectasia Mutated; *Ccnd1*-Cyclin D1; *Ccne1*-Cyclin E1; *Cdk2*-Cyclin Dependent Kinase 2; *Cdk4*-Cyclin Dependent Kinase 4; *Cdk6*-Cyclin Dependent Kinase 6; *Cdkn1a*-Cyclin Dependent Kinase Inhibitor 1A; *Cdkn1b*-Cyclin Dependent Kinase Inhibitor 1B; *GSK3β*-Glycogen Synthase Kinase 3 Beta; *Pdpk1*–3-Phosphoinositide Dependent Protein Kinase 1; *Pik3ca*-Phosphatidylinositol–4,5-Bisphosphate 3-Kinase Catalytic Subunit Alpha; *Pik3cb*-Phosphatidylinositol–4,5-Bisphosphate 3-Kinase Catalytic Subunit Beta; *PTEN*-Phosphatase And Tensin Homolog Deleted On Chromosome 10; *Trp53*-Transformation Related Protein 53.
